# A never-before opportunity to strengthen investment and action on adolescent contraception, and what we must do to make full use of it

**DOI:** 10.1186/s12978-017-0347-9

**Published:** 2017-07-20

**Authors:** Venkatraman Chandra-Mouli, Pooja S. Parameshwar, Matti Parry, Catherine Lane, Gwyn Hainsworth, Sylvia Wong, Lindsay Menard-Freeman, Beth Scott, Emily Sullivan, Miles Kemplay, Lale Say

**Affiliations:** 1 Adolescent Sexual and Reproductive Health, Department of Reproductive Health and Research, World Health Organization, 20 Avenue Appia, 1211, 27 Geneva, Switzerland; 2 United States Agency for International Development, Washington, USA; 3 Bill and Melinda Gates Foundation, Seattle, USA; 4 United Nations Population Fund, New York, USA; 5 Torchlight Collective, Nashville, USA; 6 Department for International Development, Westminster, UK; 7 Family Planning 2020, Washington D.C., USA; 8 Children’s Investment Fund Foundation, London, UK

**Keywords:** Adolescent health, Adolescent pregnancy, Contraceptive availability, Contraceptive distribution, Adolescent health services, Sustainable development goals, Family planning, Sexual health, Reproductive health

## Abstract

**Background:**

Increasingly, the health and rights of adolescents are being recognized and prioritized on the global agenda. This presents us with a “never-before” opportunity to address adolescent contraception. This is timely, as there are enormous numbers of adolescents who are currently unable to obtain and use contraceptives. From research evidence and programmatic experience, it is clear that we need to do things differently to meet their needs/fulfil their rights.

**Main body:**

In this commentary, we call for action in several key areas to address adolescents’ persistent inability to obtain and use contraceptives. We must move away from one-size-fits-all approaches, from a ‘condoms-only’ mind set, from separate services for adolescents, from ignoring the appeal of pharmacies and shops, and from one-off-training to make health workers adolescent friendly. Our efforts to expand access to quality contraceptive services to adolescents must be combined with efforts to build their desire and ability to use them, and to do so consistently. In order for these changes to be made, action must be taken on several levels. This includes the formulation of sound national policies and strategies, robust programme implementation with monitoring, regular programmatic reviews, and implementation research. Further, high-quality collection, analysis, and dissemination of data must underlie all of our efforts. As we move ahead, we must also recognize and draw lessons from positive examples of large scale and sustained programmes in countries that have led the way in increasing contraceptive use by adolescents.

**Conclusion:**

This unprecedented moment in history gives us a real opportunity to bring about transformational change, particularly when there is so much at stake.

## Background

In 2012, at the London Summit on Family Planning, representatives of more than 20 countries, the research community, the private sector, and multi−/bi-lateral organizations, donors, and foundations came together and forged an agreement to provide modern contraception to an additional 120 million girls and women in 69 of the world’s poorest countries by 2020. Over four years later, important progress has been made, as reported by Family Planning 2020 (FP2020), the partnership established following the London Summit.

This includes the finding that of the 300 million women and girls using modern contraception in 2016 across the 69 FP2020 focus countries, more than 30 million women and girls were added since 2012 [1]. However, there still remains a huge gap, especially in relation to meeting the contraceptive needs of adolescents. A key priority area in the upcoming 2017 Family Planning Summit includes more focused attention to the family planning and contraceptive needs of adolescents [2], recognizing that progress in this group has been slow and inconsistent. This presents a timely and much-needed opportunity to prioritize the needs and rights of adolescents.

### What is this ‘never-before’ opportunity?

While the Millennium Development Goals (MDGs) contributed to substantial progress in a number of areas, they did not pay explicit attention to adolescents. One of the key themes of the Sustainable Development Goals (SDGs) is to leave no one behind. The SDGs recognize adolescents (individuals aged 10–19 years) as a previously neglected group who must be addressed [3]. The updated Global Strategy for Women’s, Children’s and Adolescents’ Health also emphasizes the importance of adolescents. As the former United Nations Secretary General Ban Ki-moon said in his foreword to the document, “The updated Global Strategy includes adolescents because they are central to everything we want to achieve, and to the overall success of the 2030 Agenda. By helping adolescents realize their rights to health, wellbeing, education and full and equal participation in society, we are equipping them to attain their full participation as adults.”^1^

The 2016 Lancet Commission report on adolescents and wellbeing reiterates the triple dividend of investing in adolescents: for adolescents now, for their future adult lives, and for their children [4]. It also emphasizes that investment in their health will enable adolescents to become healthy adults who are equipped to contribute positively to society. The Commission has recently presented modeling data showing high benefits-to-cost ratios for investments in adolescent health [5]. This is also reflected in the priorities of donors, where mechanisms such as the Global Fund to Fight AIDS, Tuberculosis and Malaria and the Global Financing Facility are raising their investments in adolescent health. Clearly, we are at a ‘never-before’ moment for greater global and national attention to adolescent health and development, as noted in the recently published WHO guidance document: “Global accelerated action for the health of adolescents (AA-HA!)” [6].

One area that is in the spotlight is the need to improve adolescent access to and use of contraception; FP2020 has now made adolescent contraception a key priority. The partnership’s midpoint review, “Momentum at the Midpoint,” emphasized accountability, partnerships, and youth as key areas of focus in moving forward [1]. Specifically, the Midpoint report stated, “In order to meet the diverse needs of youth and adolescents, countries and stakeholders must examine their policies and programmes, and develop a process of evaluation and reevaluation that genuinely reflects a youth perspective, and implement evidence-based programmes that work” [1].

This ‘never-before’ moment for action to increase adolescent contraceptive use takes into account three critical factors.

- First, there is a growing recognition of the widespread unmet contraceptive needs of sexually active adolescents (both in and out of union), and the enormous health, social, and economic consequences of low levels of use. Millions of adolescents who wish to postpone or space childbearing are currently not using an effective method of contraception. The Guttmacher Institute estimates that 38 of the 252 million adolescent women aged 15 to 19 living in developing regions are sexually active, and do not wish to have a child in the next two years [7]. Among these adolescents, 23 million have an unmet need for modern contraception. This has enormous individual and social repercussions. The data modeled by Darroch et al. clearly underlines the potential demographic and public health gains of ensuring access and uptake of modern contraception by adolescents. “Meeting the unmet need for modern contraception of women aged 15-19 would reduce unintended pregnancies among this group by 6 million annually. That would mean averting 2.1 million unplanned births, 3.2 million abortions and 5,600 maternal deaths… The dramatic reduction in unintended pregnancies would spare women and their families the adverse consequences of early childbearing, reap savings in maternal and child health care, and boost women’s education and economic prospects” [7].
- Secondly, there is a stronger awareness that simply increasing the overall availability of contraception does not necessarily result in increased uptake of contraceptives by adolescents. Even where contraceptive use increases, adolescent use does not increase as much as among other age groups. Data from Bangladesh and Egypt illustrate this. In Bangladesh, adolescents aged 15 to 19 exhibit the lowest contraception utilization rates compared to older women [DHS, Bangladesh], while in Egypt, the youngest adolescents, aged 15 to 19, have the lowest levels of contraceptive use compared to older women [DHS, Egypt]. Among married 15 to 19 year olds in Egypt, 74% do not use any contraceptives [DHS, Egypt].^2^
- Thirdly, even when adolescents do have access to and begin using contraceptives, challenges still persist, including higher rates of discontinuation when compared to older women, with unmarried adolescents exhibiting the highest levels of discontinuation [8, 9].

In a recently published commentary on expanding adolescents’ access to a full range of contraceptive methods, including long-acting reversible contraceptives (LARCs), Fikree et al. summarize this never-before opportunity: “There is now a global window of opportunity for decisive, comprehensive, and collaborative action” [10].

### Why are adolescents still unable to obtain and use contraceptives?

In many places, laws and policies prevent provision of contraception based on age or marital status. Further, health workers do not provide contraceptives to adolescents due to their own personal beliefs and biases (or misinformation about laws and policies). Further, adolescents may often be unaware of where (or when) contraceptives are available, unable to afford them, or unable to easily access a contraceptive service-delivery point. Barriers such as inaccessible service locations and cost impact everyone’s ability to obtain contraception. However, these barriers disproportionally affect adolescents, as they often have limited ability to move around and financial autonomy to pay for service fees and transport. Even when adolescents are able to access contraceptives, they may be reluctant to admit that they are sexually active or simply be embarrassed to ask for contraception [11].

Focusing on access alone however, is simply not enough. The data clearly reflect that even when adolescents can obtain contraceptives, numerous factors prevent their use/consistent use. Pressure to have children, stigma surrounding non-marital sexual activity and/or contraceptive use, and fear of side effects are a few of the challenges hindering the uptake of contraceptives by adolescents [11]. Even when used, they may be used incorrectly [11]. In addition, discontinuation is a significant challenge particularly among adolescents. A number of overlapping factors can contribute to discontinuation, including side effects, hesitation to go back and seek contraceptives because of negative first experiences with health workers/health systems, changing reproductive needs (such as a partner going away for work), and changing reproductive intentions [9, 11].

An analysis of publically available data on contraceptive use by WHO clearly shows that contraception utilization, specifically by married adolescents, is appallingly low in many low and middle-income countries (LMICs). For instance, in Nigeria, 97.9% of adolescents in union aged 15 to 19 are not using any a method of contraception [12].

There is increasing attention to adolescents living with HIV, as illustrated in the WHO “Consolidated guideline on sexual and reproductive health and rights of women living with HIV” [13]. However, the contraceptive needs of many other groups of adolescents, such as those who are disabled and those in humanitarian crises are generally not acknowledged.

Barriers to contraceptive uptake vary by the situation of each adolescent. Married adolescents may not want to use contraceptives as they can face extreme pressure to bear children once in union. Unmarried adolescents may face significant stigma and judgment when seeking contraceptives. Both groups of adolescents, married and unmarried, may fundamentally not have the power to exert agency over the choice and timing of contraceptives.

### What do we need to do – or do differently – to increase correct and consistent contraceptive use by adolescents?

Evidence from reviews and studies that fed into the WHO Guidelines “Preventing Early Pregnancy and Poor Reproductive Outcomes Among Adolescents” as well as subsequent systematic reviews point to the factors that contribute to improved contraceptive access to adolescents [14–16]. For planning purposes, it is useful to conceptualize these factors into those that are supply and demand, as some frameworks have done [17, 18].

Demand-side objectives take into account the desire and agency to use contraception, while supply-side objectives address adolescents’ access to quality contraceptive services when they need them (Table 1) [18]. The International Center for Research on Women (ICRW) framework lists three demand-side and two supply-side objectives. The demand-side objectives include creating the desire to avoid, delay, space or limit childbearing; creating desire to use contraception; and building the agency to use contraception (Table 1) [18]*.* The supply-side objectives include increasing access to contraceptive services and providing adolescent-friendly services. This focus on both demand- and supply-side factors that are more responsive to adolescents’ needs is consistent with evidence from systematic reviews that show demand creation activities combined with direct provision of methods have the potential of increasing contraceptive uptake among adolescents, especially when implemented with fidelity to what works [15, 16, 19–21].

**Table 1:** Barriers and potential approaches to increasing demand for and supply of contraception among adolescents

Objective	Barriers	Successful programme approaches	Examples
Demand for contraception			
Desire to avoid, delay, space or limit child bearing	Gendered roles (expectations to be a mother, wife), need to prove fertility, religious values, path to adulthood	Enhance the acceptability of avoiding, delaying, spacing, and limiting childbearing	Conditional cash transfers have transformed life trajectories of girls in Mexico and Malawi [59, 60]
Desire to use contraception	Stigma, taboos (communication and cultural), lack of understanding (fear of side effects)	Improve the understanding of contraceptive methods and sexual and reproductive health (SRH)	Life skills education and vocational training programmes in Uganda and India have been shown to increase contraceptive use [61, 62] Working with influential family members in India helped build support and overcome resistance [63]
Agency to use contraception	Early marriage, family pressure, sexual coercion/violence, limited decision-making autonomy and power	Increase agency for girls and women to exert agency and make their own decisions	Engaging adolescents directly and their communities in Bangladesh and India has been shown to improve girls’ agency and to prevent early marriage [64, 65]
Supply of contraception			
Access to contraceptive services	Lack of awareness of services, inaccessible location, inconvenient operating hours, costs, wait times	Expand access to contraceptive services through various channels	Community-based outreach involving provision of information and services through the national Health Extension Programme (HEP) led to remarkable improvements in uptake of modern contraception among adolescents in Ethiopia [54]
Provision of adolescent-friendly services	Lack of provider sensitivity, provider reluctance to offer contraceptives to adolescents/bias, gender biases, lack of privacy/confidentiality, contraceptives unavailable or out of stock	Increase provision of high-quality, youth-friendly services for adolescents, tailored to meet adolescents’ needs	Making services responsive to the needs of adolescents has been shown to improve contraceptive use thereby preventing first pregnancies in China and repeat pregnancies in Kenya. [24, 66] Evidence from studies and projects has been applied at scale in Colombia, and Estonia [57, 67]

*Source:* Adapted from ICRW [18]

The demand for and supply of contraceptives to adolescents are also influenced by contextual factors that require appropriate programmatic responses (see Table 1). For instance, in contexts where early childbearing within or outside marriage/union is socially acceptable or encouraged, many-but not all-pregnancies are likely to be intended and interventions that aim to increase contraceptive knowledge and availability to prevent unintended pregnancy would have little effect on their own [22]. Rather, initiatives that address systematic poverty and social disadvantage, including gender inequality, can improve girls' ability to negotiate their relationships, contraception, and fertility decisions. This includes initiatives that seek to tackle child, early, and forced marriage, which contributes to adolescent childbearing [22]. In contexts where there is enormous pressure to bear children soon after marriage, key programmatic objectives should be to delay first births through social norm change and to provide contraception to (the minority of) couples who want them, and support norm change and support adolescent girls to space their subsequent births.

Let us now examine what we need to do differently to improve access to and provision of contraceptive services to adolescents.

Firstly, we must move from a one-size-fits-all approach to one that responds to the varying needs of different groups of adolescents. Programmes have addressed adolescents as a homogenous group. We must now understand and respond to the differing needs of different groups of adolescents, such as sexually active younger adolescents (those below 15 years of age), those who in union/in union including newlyweds, first-time parents, and those living HIV. Deliberate, targeted approaches will be needed to reach out to those adolescents such as those who are physically or mentally disabled whose sexual and reproductive needs are not acknowledged, leave alone addressed.

Data from Tanzania and Nigeria clearly illustrate the pressing need to reach adolescents who are married/in union. In Tanzania, while 60.3% of unmarried sexually active 15 to 19 year old adolescent girls are not using contraceptives, 85.1% of those who are in union are not doing so [12]. The figures from Nigeria are even more striking. Here, 44.9% of unmarried, sexually active 15 to 19 year old adolescent girls are not using contraception, while 97.9% of adolescents in union are not doing so [12].

Secondly, we must expand the range of contraceptive choices offered to adolescents from ‘condoms only’ to the full range of methods. The only contraceptives that most health workers advise adolescents to use are condoms – or occasionally pills – especially to unmarried adolescents. This can be because they often believe that long-acting hormonal contraceptives and intrauterine devices hinder fertility and should be offered only after the first child is born. We must change health workers’ beliefs, attitudes, and practices in this crucial area because there is no medical reason to deny adolescents any method [23].

As shown in Kenya and Ethiopia, training and supporting health workers and educating consumers can increase both the provision and uptake in LARCs [24, 25]. In Ethiopia specifically, the Evidence to Action (E2A) Project demonstrated that when health workers are trained and supported to provide LARCs, adolescents are more likely to utilize them [26].

Thirdly, we must move away from separate health services for adolescents, and instead make existing health services that already serve adolescents, to some extent, more adolescent friendly e.g. antenatal clinics, postnatal clinics, STI/HIV clinics, and as extensions to under-five clinics. Using programming principles and practices that we have learned e.g. providing non-judgmental care and ensuring confidentiality are likely to make these services responsive to adolescents’ needs and preferences. Meaningfully engaging adolescents in designing and implementing services is both a useful means to the end of increasing uptake and an end in itself. Finally, given that separate services have been shown to be difficult to scale up and sustain in resource-constrained settings, focusing on strengthening existing services is a pragmatic approach to reach large numbers of adolescents [27].

Community health outreach workers and volunteers can also be mobilized to reach out to adolescents in the community who may be unable or unwilling to seek contraceptives at health facilities. For example, in Madagascar, UNFPA and Marie Stopes implemented an e-voucher programme to address the unmet need for family planning among adolescents. As part of the programme, young outreach workers disseminated information, distributed e-vouchers, and provided clinic referrals for a range of contraceptives including LARCs [25]. Results from Madagascar indicate that this combination of strategies can promote a wider range of contraceptive uptake.

Fourthly, we must work more actively with pharmacies and shops to expand contraceptive access and uptake, as our current focus on public health facilities alone does not fully reflect where adolescents obtain their contraceptives. Data from Tanzania and India illustrate the importance of pharmacies and shops as sources of contraceptives to adolescents. In Tanzania, many unmarried, sexually active adolescents obtain contraception from a shop (44.6%) or from friends (11.2%), and many adolescents who are married/in union get it from a government facility (53.0%) or a shop (31.8%) [12]. In India, most adolescents obtain contraception from the private sector, with most unmarried, sexually active adolescents obtaining contraceptives from a private facility (46.0%) or a pharmacy (44.3%) and married adolescents getting contraceptives from a pharmacy (32.3%) or a shop (27.5%) [12]. Although Tanzania has no legal or policy barriers that prevent unmarried adolescents from obtaining contraceptives from government facilities, as compared to India which does have such restrictions, both countries demonstrate high rates of pharmacy utilization among both married and unmarried adolescents. Pharmacies may offer adolescents greater convenience, longer operating hours, accessible locations, and ease and speed of service including access to oral and emergency contraceptives [28]. However, the information provided/advice given may not always be accurate. The preference for pharmacies is especially manifest in contexts where obtaining contraceptives from government health facilities may be difficult due to restrictive laws, health worker bias, or because young women do not want their partners/families to know. A recent study in Nepal showed that emergency contraceptive pills were “popular among young (less than 25 years old) and educated women” who preferred to obtain them from private pharmacies [29].

Lastly, we must move from one-off in-service training for a handful of providers to a package of actions to ensure that all levels of health workers, including support staff, respond to adolescent clients effectively and with sensitivity. One-off training programmes, even if well conducted, have limited and short-lived effects. A comprehensive package of actions is needed: clear job descriptions grounded in quality standards (as proposed by WHO’s “Global standards for quality health care services for adolescents” [30], good quality competency-based pre and in-service training [31], desk reference tools and job aids, supportive supervision, and collaborative learning [32]. This point is reinforced in a review by Dieleman et al. which ascertains the strength of combined approaches. “Combined interventions of participatory, interactive training, job aids, and strengthening health systems can be successful in improving health workers’ performance” [33].

Alongside our efforts to improve the quality and expand the availability of contraceptive services, we must provide adolescents with comprehensive sexuality education (CSE) in an age and developmentally-appropriate manner. This is because knowledge gaps and misconceptions on contraception both reduce uptake and increase discontinuation of contraceptive use. This is clearly illustrated by WHO’s analysis of contraceptive use in a number of countries. For example, in Ghana, 37.4% of unmarried, sexually active adolescent girls and 20.5% of adolescent girls in union do not use contraceptives due to fear of side effects or health concerns. Similar patterns are reflected in Jordan (29.5% married), the Philippines (26.5% married), Haiti (26.1% unmarried; 26.3% married), Tanzania (18.4% unmarried; 15.0% married), Kenya (12.8% unmarried; 13.9% married), and Liberia (19.1% unmarried; 7.5% married) [12]. While this section addresses the provision of contraceptive information and services, it is important to ensure strong linkages to other sexual and reproductive health services including antenatal, delivery and post natal care, safe abortion care, STI and HIV/AIDS care, and to other health services e.g. mental health.

### Expanding access to quality contraceptive services to adolescents with equity: What do we want to see in countries?

i. *Develop national laws and policies that require health workers to provide contraceptive services to adolescents without restrictions, and communicate them widely*

National laws and policies vary a great deal in terms of how enabling they are, as illustrated by a recent analysis by the Population Reference Bureau (PRB) [34]. PRB analysed country policies on family planning services using several indicators. In relation to three criteria, external authorization, age restrictions, and marital status restrictions, PRB found that Tanzania, for instance, meets all three criteria, Nigeria meets two criteria, and the Democratic Republic of Congo (DRC) meets none of the three criteria. More specifically, Tanzania’s policies do not require external authorization; nor do they restrict provision by age or marital status. Nigeria’s policies require external authorization, but do not restrict provision by age or marital status, although these latter two policies contained “room for improvement,” according to PRB. Finally, the policies of DRC require external authorization and do restrict by age and marital status [34].

Strengths and weaknesses of national contraceptive policies and strategies can be examined in more detail. In one study analysing South Africa’s contraceptive policies and strategies using the nine recommendations in WHO’s guidelines on “Ensuring human rights in the provision of contraceptive information and services” [35] as an analytic lens, some gaps were identified in availability, informed decision-making, and adolescent engagement in programme development [36]. However, South Africa’s normative guidance as a whole contained substantially more strengths than weaknesses. Similar results are soon to be published in a study analysing national contraception laws, policies, and regulations from Paraguay. This analysis found that Paraguay’s normative guidance was grounded in human rights principles, but could be strengthened in relation to both the quality of contraceptive provision and access [37]. Analysing national level policies with credible benchmarks can point to areas that need to be addressed to make them more enabling. While the above discussion focuses on access to contraceptive information and services, the very same principles apply to other sexual and reproductive health services such as safe abortion and HIV testing and counselling.

ii. *Design sound national adolescent sexual and reproductive health (SRH) strategies that contain evidence-based and context-specific packages of interventions, budgets to deliver the package, and indicators to track progress that are disaggregated by age, sex, and socio-economic status*

Policies provide the basis for action, but action cannot be achieved without sound national strategies to operationalize them. National strategies must be based on the scale of the problem and the factors that contribute to it. They must choose interventions that address local determinants, are feasible to implement, and have demonstrated effectiveness. Alongside strategy development, indicators to assess inputs, processes, and outputs must also be incorporated. In developing the Global Financing Facility investment case for Liberia, a consultative approach involving government bodies, nongovernment organizations and international development partners was employed. The outputs of this exercise are shown in Table 2.

iii. *Implement strategies with fidelity and careful monitoring, through functional systems and with the participation of civil society groups (including networks of youth organizations)*

**Table 2:** Intervention and monitoring framework on the prevention of early and unintended pregnancy and sexually transmitted infections including HIV in Liberia

Interventions to address the determinants	Factors that contribute to the adolescent behaviours	Adolescent behaviours most directly related to these health outcomes	Health outcomes
Population-wide actions:	Individual	Unprotected sexual activity Early initiation of sexual activity Early marriage	Early pregnancy Sexually transmitted infections, including HIV
1. Improve community awareness about adolescent sexuality andsupport for protecting adolescents with sexuality education and sexual and reproductive health services, notably contraception and HIV testing and counselling. 2. Improve access the contraceptive information and services and HIV testing and counselling services to all segments of the population.	Adolescents have many knowledge gaps about sexuality and reproduction and how to avoid problems. They have many misconceptions about contraceptives.		
	Immediate environment		
	Adolescents are not able to get sexuality education at home, school or elsewhere in the community. Adolescents are not able to obtain contraceptives from government facilities and cannot afford them from private providers. They are under pressure to have sex early from peers and from adults		
Adolescent-specific actions:			
1.Educate boys and girls about sexuality and reproduction. 2.Build individual and social assets of adolescent girls to avoid from choosing child marriage/to being forced into child marriage. 3.Improve access and uptake of contraception through government clinics and through complementary approaches community outreach, social marketing and commercialsales. 4.Improve access to HIV testing and counselling and links to HIV-related care.			
	Wider environment		
	Social norms do not acknowledge adolescent sexuality and are not supportive of providing adolescents with sexuality education and contraception. There are few educational and employment opportunities especially for girls. Traditions and economic constraints pressure families to have their daughters married early.		
Indicators and means of verification			
Quality and coverage of school and community based education and health service provision (Source: Implementation reports and assessments)	Proportion of adolescent boys and girls who are knowledgeable about contraception and know where they can get them (Source: Surveys)	Proportions of adolescent girls who report using modern contraception and boys who report using condoms (Source: Surveys)	Data on adolescent fertility, and prevalence of STI/HIV disaggregated by age and sex (Source: Surveys)

*Source:* Liberia 2016–2020 Investment Case for Reproductive, Maternal, Newborn, Child, and Adolescent Health (RMNCAH) [68]

Enabling national policies that feed into sound strategies for implementation and monitoring are important. But the real barrier in many countries is the strategy implementation gap. To ensure fidelity of implementation, it is essential to have functioning systems and technical expertise drawing upon a range of players. This is powerfully reinforced by the Health Policy Plus project in their analysis of family planning policies for young people in Guatemala, Malawi, and Nepal, “All three countries have largely recognized the problems facing adolescent women and are trying to meet their family planning needs. These countries have many of the right policies already in place.” However, the study found that in all three countries, despite the presence of enabling policies, adolescents found it difficult to obtain contraceptives. Thus, in line with the recommendations of the authors of the report, while it is important to add to or refine existing policies, what matters most is how well countries implement available policies [38].

To ensure fidelity of implementation, it is essential to put systems in place. Mozambique provides an excellent example of a country which designed and implemented systems to ensure the engagement with and coordination among the Ministries of Health, Education, and Youth and Sport at the national, provincial and district levels [39]. The objectives of the programme – later branded Geracao Biz (Busy Generation) – were: ‘To improve Adolescent Sexual and Reproductive Health, including a reduction in the incidence of early and unintended pregnancy, Sexually Transmitted Infections and Human Immuno-Deficiency Virus infections, through activities that equip young people with the knowledge, skills and services needed for positive behaviour change.’ From the outset, Geracao Biz was a truly multisectoral programme; government staff from the three sectors worked with community-based and youth-serving organizations, as well as young people, to deliver three complementary interventions: youth friendly clinical services, school-based education, and community-based outreach. A strong coordination mechanism facilitated collaboration at all levels of the system, with active involvement of young people.

The Udaan project in Jharkhand state, India demonstrated how civil society bodies with expertise in adolescent health supported a government-led school-based adolescent health education programme. The Centre for Catalyzing Change (C3), an indigenous nongovernmental organization, worked with the government to develop a curriculum, build and refresh teacher skills, and monitor/evaluate the programme’s effects. It also supported the government to build community support and deal with resistance, when this arose.

Since 2006, 1485 secondary and senior secondary schools including *Kasturba Gandhi Balika Vidyalayas* (KGBVs) across all districts in the state are being reached. Emboldened by the success of the programme in secondary and senior secondary government schools of the state, the programme has been extended to students in upper primary classes of over 100 selected government middle schools of Ranchi, Ramgarh, and Lohardaga districts as well as 203 KGBVs across the state. C3’s central role in Udaan is key to successful expansion and sustenance, when adolescent education programmes in many other states of India have not been able to run, or be implemented at scale [40].

One of the strengths of Udaan was the involvement of civil society organizations; similarly, the involvement of young people can also play a critical role in identifying needs and problems, drawing attention to them and helping find solutions. An example of this is the YP Foundation’s work with young people in Sunder Nagar Nursery, an urban slum community in New Delhi, India. The Foundation supported young leaders in auditing and mapping the safety of young people in the area using electronic tools and methods tried and tested internationally, and in engaging with the Delhi Police, the Municipal Corporation of Delhi and the Delhi Government’s elected representative to share their findings and to find solutions [41]. Indeed, the onus is on to finding ways to engage adolescents meaningfully – with ‘voice,’ ‘minds and hands’ and ‘teeth.’ Firstly, it is essential that we hear adolescents’ ‘voices’ to understand their needs and problems. Secondly, we must tap into their ‘minds and hands’ to help design, implement, and assess policies, programmes, and projects that are intended to meet their needs. Thirdly, we must put in place mechanisms to enable adolescents to hold individuals and institutions accountable for gaps and shortcomings- ‘teeth’. Adolescent ‘voice’ is important, but that alone is not enough.

iv. *Conduct periodic programme reviews to build on strengths and address weaknesses*

Periodic monitoring and evaluations of programme implementation, and reviews of the findings to assess actual against proposed effects are critical to success. An example of a successful national, multisectoral strategy to address teen pregnancy that utilized consistent programme reviews can be seen in the United Kingdom Government’s 10-Year Teenage Pregnancy Strategy (TPS) for England from 1999 to 2010 [42]. The primary goal was to reduce the under-18 birth rate by at least 50% from 1998 to 2010; by 2014, the rate had been lowered by 51% [42].

Many of the major strengths of England’s approach are attributable to the rigorous collection and utilization of data. Data and qualitative research were regularly reviewed to identify potential risk factors for early pregnancy or poor outcomes for young parents and their children. Summaries were provided to local areas to inform targeted work, and strategic actions were integrated into relevant national government programmes aimed at improving health and educational outcomes for the most disadvantaged young people. Five years into the implementation of the strategy, adolescent pregnancy rates had declined in some districts, but in others, rates were stagnant or even increasing. The government implemented a “deep dive” to examine why progress was uneven, by matching three ‘highly-performing’ districts with three demographically similar yet ‘poorly-performing’ districts. The review pointed to strong leadership and the implementation of all elements of the recommended package in the highly performing districts. Based on these findings, the government put in place more prescriptive guidance and conducted active, quarterly follow-up of less well-performing districts. These efforts extended the declines in teen pregnancy to all of the districts in the country and illustrate how national authorities can use data to inform collective action at the local level.

England’s midterm review contributed to change in the direction of the programme by pointing to gaps and areas of weakness. Similarly, a recent situation analysis in Bangladesh found that many indigenous and international organizations were using approaches that were out-dated and not always evidence-based. These efforts failed to reach unmarried adolescents with services, and were also isolated to certain geographic regions [43].

v. *Research – with an emphasis on implementation research - to answer context-specific programmatic challenges*

There is growing evidence on what works, and what does not work, in responding to the needs and problems of adolescents. However, ineffective interventions and ineffective delivery mechanisms continue to be widely implemented, resulting in several common scenarios: adolescents are not reached by interventions; interventions demonstrated to be effective are delivered ineffectively; interventions are delivered piecemeal, with exclusively micro or macro engagement; interventions are delivered in low “dosages” (short duration or intensity); popular interventions that have been shown to be ineffective for adolescents continue to be proposed [20]. This represents a waste of scarce human and financial resources and raises questions about the value of adolescent health policies and programmes.

While we must strive to apply what we already know, we must work to fill gaps in our knowledge and understanding [44]. This includes developing and testing new interventions, and testing the scalability of interventions, which have been shown to be efficacious in research studies and tightly controlled projects. Cost analysis and cost-effectiveness studies are also urgently needed. Hindin et al. outline research priorities for adolescent sexual and reproductive health in LMICs [45]. Selected questions that relate to contraception are listed in Table 3.

**Table 3:** Research priorities for contraception in adolescents Assertion

• In settings with high rates of pregnancy in adolescence, what factors protect adolescents from unwanted and/or unsafe pregnancy?	
• What strategies can delay first births among married adolescents?	
• What strategies can increase consistent and effective condom use among both male and female adolescents?	
• What barriers do health-care providers face when trying to offer contraception services to unmarried adolescents?	
• Through what mechanisms can the provision of regular and emergency contraceptives to adolescents be financed or subsidized?	

*Source:* Adapted from Hindin et al. [45]

Research on the strengths and weaknesses of policies and programmes can be useful in pointing to the reality on the ground. For example, the Guttmacher Institute has recently published assessments of the shortcomings of CSE programmes in three countries [46–48]. We need, however, to move beyond problem identification to support robust implementation research to determine the feasibility, acceptability, and effectiveness of approaches to improve the quality and coverage of programmes, building on what exists. Using policy and programmatic obstacles as starting points, implementation research employs methods to overcome identified obstacles. For example, a paper by Vanwesenbeeck et al. points to a number of programmatic challenges that were identified in the implementation of a CSE programme, “The World Starts With Me,” in 11 low-income countries in Africa and Asia. These include lack of programme fidelity, inadequate teacher skills, and contextual factors (school social climate, limited access to youth-friendly services). The authors argue that focusing on these limiting factors, rather than on tweaking the curriculum, has greater potential to increase the effectiveness of CSE programmes [49].

### We need sound data to shape and reshape our efforts

Each of the stated priorities must be informed by data. Specific data that must be collected include: rates and outcomes of adolescent pregnancies; contraceptive use and its determinants; policies and programme performance, and information on adolescent sexuality and its context. WHO’s fact sheets on adolescent contraceptive use in 58 LMICs contribute to this by providing comprehensive data on contraceptive use by marital status, types of contraceptive methods used, why contraception is not used, and where adolescents obtain contraception [12]. However, as these fact sheets were generated from Demographic and Health Surveys, they are not available for every country, and some surveys have critical gaps in data collection. For example, contraceptive usage data are only available for married adolescents in many countries with large adolescent populations including Pakistan and Indonesia [12].

While the currently available survey data clearly demonstrate the diversity of the adolescent populations, there are limitations in how they can be used. National level data represent averages and are not always applicable for local level programming. Also, the intervals between surveys mean that they cannot be used for most programme monitoring purposes. Track20 has worked to bridge the gap between surveys and programme data, with promising results [50]. However, the applicability of such efforts to adolescent populations depends on the availability of disaggregated programme data, which is currently weak, as few health information systems disaggregate contraceptive data by age.

Efforts to define global core indicators have been successful in harmonising a set of measures to monitor child and adolescent health [51, 52]. These efforts support global monitoring and coordination, but beyond quantifying needs and problems in the form of adolescent pregnancy rate or child marriage prevalence, give little information on national efforts.

Seizing the current opportunity, national efforts scaling up adolescent family planning services will have to be complemented with robust measurement of progress on all levels from inputs through process, outputs, outcomes and health impact. As discussed above, the evidence of interventions in the adolescent population shows that interventions can be effective in changing behaviours. However, the same body of evidence also shows compellingly, that despite using basic strategies with proven efficacy, many interventions are ineffective due to features in design and implementation [15, 20]. Moving forward, programmes have to be able to gather the needed information to identify these issues, and the collected data should then be aggregated, analysed and used for decision-making.

### Outstanding examples of countries moving ahead with adolescent contraception

A small and growing number of countries are showing impressive progress in expanding contraceptive use by adolescents.

Ethiopia is one example of a country that has increased contraceptive use among some groups of adolescents, as part of a programme designed to reach the population as a whole. Specifically, in 2003, Ethiopia launched a national Health Extension programme (HEP), with the aim of achieving universal health coverage. Central to HEP are the Health Extension Workers (HEWs). “Health Extension Workers are the key players in the programme. They are all female, 10th grade high school graduates, recruited from the community with the active participation of the community. They are trained for one full year and then deployed back into the community to promote health and provide services at the village level. Two HEWs are paired to serve 3,000 to 5,000 people and serve at a health post. Much of their time is devoted to home visits and outreach. Over 35,000 HEWs are recruited, trained, and deployed to the villages” [53].

From 2005 to 2011, modern family planning utilization among married women – including married adolescent women -- increased from 10.9% to 23.4% [54]. According to The World Bank report on HEP in Ethiopia, “…this high uptake in family planning is due to the contribution of the HEWs and the availability of contraceptives through the HEP” [53]. This is particularly remarkable given that married adolescents in rural areas tend to be disproportionately disempowered and typically exhibit the lowest rates of contraceptive usage.

While there were increases of contraceptive use in Ethiopia, two other countries within sub-Saharan Africa, Burkina Faso and Nigeria, demonstrated stagnation and decline [54]. Hounton et al. summarize the relationships between the three countries, further underscoring Ethiopia’s advances. “While there was no significant progress in Burkina Faso and Nigeria, the data in Ethiopia point to a significant and systematic reduction of inequalities. The narrowing of the equity gap was most notable for childbearing adolescents with no education or living in rural areas” [54].

These results were achieved through a range of actions, including supportive policies for contraceptive use for adolescents regardless of marital status or age, regional work plans and budgets, training of HEWs, and a disaggregated health management information system. Increasing adolescent contraception among married adolescents was not Ethiopia’s only achievement. In recent years, the country has also seen declines in child marriage and female genital mutilation/cutting because of progressive legislation and efforts by governmental, international, and civil society organizations [55].

Estonia is an excellent example of a successful programme that was deliberately targeted at adolescents. In the mid-1990s, Estonia scaled up grassroots SRH services to a national youth clinic network and alongside that, rolled out a widespread CSE programme in schools. Over a period of 15 years, the abortion rate decreased by 61% and fertility rate by 59% amongst adolescents aged 15 to 19 years [56]. Coupled with CSE, a network of adolescent friendly sexual health NGO clinics supported by the government facilitated an environment of concurrent positive social change [57].

There were many social, political, and economic changes in Estonia in the early 1990s that provided a fertile ground for innovation and investment. Motivation to address adolescent SRH was particularly high, given that there were high unwanted pregnancy and abortion rates, rising STI incidence, and low usage of reliable contraceptives during this time period [57].

Although Estonia was committed to the scale-up, and the environment within the country was ripe for change, a critical factor that allowed for Estonia’s success was the substantial assistance from international organizations and neighbouring countries. International Planned Parenthood Federation European Network provided a platform for Estonia and other Scandinavian countries to collaborate and share resources and insights into best practices and progress on SRH programmes.

A supportive social and political climate, demonstrated need for adolescent SRH services, a national organization representing the youth clinics, commitment of personnel, acceptance by user organizations, and sustainable funding through national health insurance are all attributed to Estonia’s success [56].

Ethiopia and Estonia represent two different national approaches to contraception – population-based and targeted, respectively. Both of these approaches, alone or in combination, may be applicable in other contexts.

## Conclusion

We have a never-before opportunity to address adolescent health. The work of FP2020 and the Family Planning Summit have placed adolescent contraception high on the global agenda.

Our understanding of the complex web of factors that help and hinder adolescent contraceptive uptake and use has grown substantially since the International Conference on Population and Development pointed to this in 1994. Today, we have a much better sense of what needs to be done to meet the needs of adolescents, and to enable them to realise their rights and exert agency over their sexual and reproductive decisions.

Much of our evidence base comes from small-scale research studies and projects. We have reached a limit of what we can learn from them. The big questions now revolve around how to orchestrate and sustain large-scale implementation, how to respond to the diverse needs of different groups of adolescents, and how to involve adolescents and young people in these processes. These questions can only be resolved if we support countries to scale up adolescent contraceptive information and service provision with quality and equity, and learn through this process.

Some countries, such as Ethiopia and Estonia, are already moving forward. We need to recognize and celebrate their successes and share them widely. But the real challenge facing us is to stimulate and support similar progress in other countries across the world.

We are currently two years into the SDGs. If we are unable to show that countries are beginning to make progress in adolescent contraception in the next few years, we run a real risk of losing the interest and support we currently enjoy. The focus of the global community could well shift onto other pressing issues in the health and development agenda. Most importantly, we will fail to deliver on the promises we have made to current and future generations of adolescents.

We know where to go, and have a clear idea of the next steps. While we still have much to learn, we know that sustainable implementation of large-scale programmes requires courageous leadership, good science, and strong management. We have never had a better opportunity to act than the one we have at hand.

## Abbreviations

CSE: Comprehensive sexuality education
FP: Family planning
ICRW: International Center for Research on Women
LARC: Long-acting reversible contraceptive
LMIC: Low and middle-income countries
MDGs: Millennium Development Goals
SDGs: Sustainable Development Goals
SRH: Sexual and reproductive health
UNFPA: United Nations Population Fund
WHO: World Health Organization

## Footnotes

1 To monitor programmes, and especially their outcomes and impact, the Global Strategy for Women’s, Children’s and Adolescents’ Health has 60 indicators that are either adolescent specific (e.g. adolescent mortality rate) or include adolescents (e.g. experience of sexual violence).

2 Unmet need for family planning is a relatively complex indicator as it requires simultaneously measuring sexual activity, fertility preference and fertility as discussed by Cleland et al. [58]. The main concern in adolescent populations is the measurement and definition of sexual activity, especially in unmarried adolescents.
